# Therapeutic Potential of Adjuvant Stereotactic Body Radiotherapy for Gallbladder Cancer

**DOI:** 10.7759/cureus.299

**Published:** 2015-08-10

**Authors:** Anand Mahadevan, Nergiz Dagoglu, Jennifer F Tseng, Khalid Khawaja, Amy Evenson

**Affiliations:** 1 Department of Radiation Therapy, Beth Israel Deaconess Medical Center; 2 Department of Radiation Oncology, Istanbul University Istanbul Medical Faculty; 3 Department of Surgery, Beth Israel Deaconess Medical Center

**Keywords:** gallbladder cancer, adjuvant, sbrt

## Abstract

Surgical treatment remains the only curative treatment for gallbladder cancer. However, even after liver resection, locoregional failure seems to be a significant problem. While there is no Level I evidence, multiple studies have shown benefit for adjuvant radiation in high-risk patients. After extensive liver resection, tolerance to conventional chemoradiation may be limited by potential liver toxicity. Stereotactic body radiotherapy has been used safely and effectively in hepatobiliary malignancies. We present a case report, highlighting the potential therapeutic role of adjuvant stereotactic body radiotherapy (SBRT) for gallbladder cancer.

## Introduction

Gallbladder cancer (GBC) is a rare malignancy. Its incidence varies widely geographically with an age-standardized incidence rate of 1.7 in the US to as high as 10.4 in Chile [1]. In 2014, there were 10,650 gallbladder and extrahepatic bile duct cancers in the US with 3,630 deaths [2]. Surgical resection offers the only curative treatment for gall bladder cancer. While gallbladder cancers can be incidentally detected after cholecystectomy, the majority of patients present with invasive liver disease [3]. Even after organ-confined disease, local recurrence, in up to 65% of patients, is a significant cause of morbidity and mortality [4]. This has led to the use of liver resections to improve outcome in gallbladder confined disease [5] and the use of adjuvant chemotherapy and chemoradiation [6-8].

Despite the potential role of postoperative RT [9-11], its limitation remains radiation-induced liver disease, which develops in 5% to 10% of patients exposed to an excess of 30 to 35 Gy of radiation, depending on irradiated liver volume and hepatic functional reserve [12]. This is particularly true after hepatic resection with limited reserve. Very little data exists describing the risk of hepatobiliary toxicity for lesions near the central hepatobiliary area around the porta hepatis [13].

Stereotactic body radiotherapy (SBRT) enables the delivery of effective doses of radiation to tumors with steep dose gradients minimizing doses to the surrounding normal tissues. It has been successfully used in hepatobiliary and pancreatic malignancies. Moreover, short courses of SBRT avoids delays or interruption of systemic therapy [14-16].

We present a report of two cases where SBRT was used as postoperative radiation for gallbladder cancer in the adjuvant setting.

## Case presentation

This study was reviewed and approved by the Dana-Farber Harvard Cancer Center Institutional Review Board (DFHCC 09-451). Informed patient consent was obtained from all patients in this report.

### Case 1

A 70-year-old female presented with a one-month history of right upper quadrant abdominal pain. She noted a 20-pound weight loss and significant loss of appetite over the previous three months. On physical examination, the liver was palpable 4 cm below the right costal margin and was firm and tender. She underwent an ultrasound of the liver that demonstrated a large mass in the right lobe of the liver that was heterogeneous, predominantly hypoechoic, with internal vascularity and measuring 10.5 x 7.4 x 12.2 cm. There was no intra- or extrahepatic biliary ductal dilatation. She then underwent an MRI of the abdomen that demonstrated a mass within the anterior segments of the right lobe of the liver measuring 7.1 x 4.6 x 5.2 cm that had heterogeneous decreased signal T1 images with slightly increased signal on T2 imaging. She underwent an upper GI endoscopy and colonoscopy that demonstrated no evidence of primary tumors. Her liver function tests revealed an AST 23, ALT 24, alkaline phosphatase 169, total bilirubin 0.5, CEA less than 0.5, and CA 19-9 34.

The patient received induction gemcitabine and capecitabine chemotherapy and portal vein embolization to induce left lobe hypertrophy. Restaging scans showed no evidence of metastasis and significant response with decreasing size of the lesion. She then underwent a right hepatic resection of the gallbladder fossa and the involved liver. Fiducial seeds were placed in the liver bed. It is our institutional practice to place fiducials in the liver tumor resection bed in all liver tumor resections at the time of surgery. The pathology showed that the patient had a poorly differentiated adenocarcinoma in the gallbladder with secondary involvement of the liver making this T3 disease. The cauterized liver margin was involved with invasive carcinoma; perineural invasion was absent. None of the lymph nodes was involved. She was thought to be a candidate for adjuvant postoperative radiation therapy since she had high-risk factors of a positive margin, T3 stage, and high grade. Because she had little residual liver, SBRT was advised.

The planning target volume was a 1.5 cm deep segment of the liver resection margin, which included the fiducial seeds. She received 24 Gy in three consecutive fractions to the liver resection bed. She completed the therapy with minimal side-effects, including fatigue and tiredness for a week and was well at her last follow-up 24 months later.

**Figure 1:**
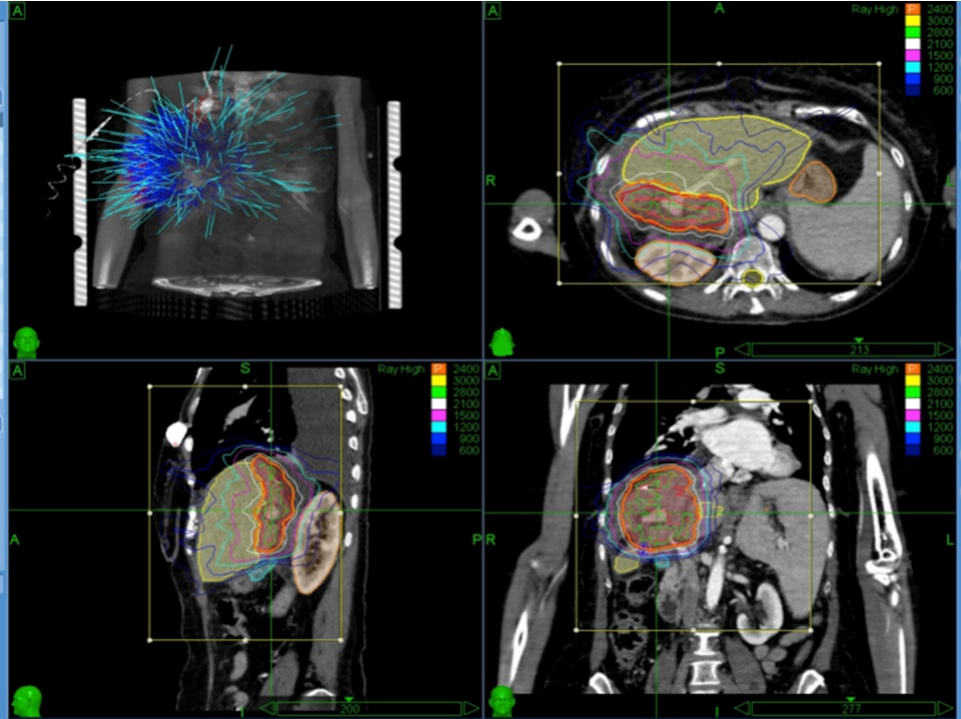
Representative Treatment Plan

A representative treatment plan is shown in Figure 1. The treatment prescribed to the 79% isodose line using the CyberKnife^TM^ technique with Synchrony respiratory tracking. The max dose was 30.4 Gy and the V21 (volume of residual liver receiving 21 Gy) and V15 (volume of residual normal liver receiving 15 Gy) was 11% and 21%, respectively. The maximum dose received by the < 1 cc of bowel was 17 Gy in three fractions.

### Case 2

A 77-year-old gentleman, known to have bladder cancer along with cardiac disease, had staging surveillance scans by his urologist. The CT showed focal gallbladder wall thickening in the fundus with enhancement consistent with probably what was thought to be a 2.3 x 1.6 x 2.6 cm tumor mass suspicious for primary gallbladder cancer. The tumor marker CA19-9 was elevated at 287. He had a cholecystectomy with partial hepatectomy and resection of segment IVB and V as well as the removal of portal nodes. Fiducial seeds were placed in the tumor bed during surgery. The pathology showed a moderately differentiated carcinoma of 2.8 cm in the gallbladder invading the perimuscular connective tissues. There was no evidence of regional metastasis. There was a perineural invasion, but no evidence of any venous invasion. He developed cardiac toxicity with adjuvant capecitabine. In view of his high-risk disease, age, and co-morbidities, he was advised to undergo adjuvant radiation with SBRT followed by gemcitabine chemotherapy. At his last follow-up 74 months after surgery, he was still free of disease.

The planning target volume and the treatment technique was similar to the previous patient. He received 24 Gy in three fractions to the 68% isodose line with a maximum dose of 35.3 and the  V21 and V15 - 9% and 17%. The maximum dose to < 1 cc of bowel was 10 Gy in three fractions.

## Discussion

Complete surgical resection remains the only potential curative treatment for GBC [17]. Additional liver resections improve outcomes in incidentally detected cancer and in organ-confined and locally advanced disease [4, 18-19]. However, even after organ-confined disease, locoregional recurrence is a significant problem [4, 20]. The high incidence of locoregional recurrence in patients with GBC makes postoperative radiotherapy (RT) a rational option. The benefit of adjuvant RT or chemoradiation has not been tested in randomized studies. However, attempts to define the role of adjuvant RT have been made by single and multi-institutional studies [9, 11, 21-22] and analysis of national databases. Mojica, et al. analyzed 3,187 patients from the SEER (Surveillance, Epidemiology and End Results of the National Cancer Institute) population database of whom 17% were treated with adjuvant radiotherapy [23]. For patients presenting with locally advanced disease and lymph node involvement, an increase in median survival with adjuvant treatment (14 versus 8 months, p < 0.001) was observed. Hyder, et al. analyzed a sample of 5,011 patients (18% received adjuvant RT) and reported that RT was associated with an improved short-term survival but the benefit of RT seemed to dissipate after 12 months because there was no difference in five-year survival [24]. This highlights the role for systemic therapy in controlling metastatic disease [8, 25]. It should be noted that in neither of the studies was margin status evaluated. A recent systematic review and meta-analysis on biliary tract cancers included six studies addressing the role of adjuvant treatments in GBC. However, due to the rarity of this disease, there is no Class I evidence from randomized clinical trials supporting the role of adjuvant radiation or chemoradiation. The National Comprehensive Cancer Network Guidelines for GBC suggest adjuvant chemoradiation or chemotherapy alone, recognizing, however, that limited data exist to define a standard regimen.

Several investigators have used multivariate analysis to determine useful prognostic factors for recurrence of gallbladder carcinoma after surgical resection to identify patients who might benefit from adjuvant therapy. Margin positive (R1 resection) patients derived the clearest survival benefit from the use of adjuvant therapies [26-27]. It would also appear to benefit patients with high T stage and positive nodes [28]. Others have developed multivariate predictive models [25] and nomograms [29] for risk assessment. By these reports, other potentially significant factors include tumor grade and perineural invasion.

In the present report, our patients carried high-risk features of a positive margin, liver invasion, and high grade and were in a subgroup where studies favored adjuvant treatment. However, they had small residual liver after their liver resections. This places them at a high risk of radiation-induced liver injury. It is known that radiation-induced liver disease develops in 6% to 66% of patients exposed to an excess of 30 to 35 Gy of radiation, depending on irradiated liver volume and hepatic functional reserve [28]. These patients may face RILD (radiation-induced liver damage) starting with the classic triad of ascites, hepatomegaly, and elevated liver enzymes. The majority of patients recover completely in three to five months, while some progress towards a chronic stage, with worsening liver fibrosis and failure, developing fulminant hepatic failure [12].

To reduce the radiation to remnant liver and the risk of RILD, SBRT was proposed as adjuvant treatment in our patients. SBRT has been reported to be used effectively and safely for the treatment of hepatobiliary and pancreatic cancers in the setting of both primary and adjuvant treatment [14-16]. Effective radiation therapy can be delivered to the tolerance of the adjacent critical normal tissue structures. A report from Korea also addressed the use of SBRT in unresectable gallbladder cancer in a case series that included four GBC patients [30].

## Conclusions

SBRT can potentially be safely delivered to a planned postopertaive liver resection target volume as decribed. This first report of use of SBRT in the postoperative adjuvant setting may pave the way of safe and effective adjuvant radiation for gallbladder cancer in future studies.

## Appendices

CONSENT

Written informed consent was obtained from the patients for publication of their treatment with accompanying images accompanying images. A copy of the written consent is available for review by the Editor-in-Chief of this Journal.

COMPETING INTERESTS

The authors declare that they have no competing interests.

AUTHORS CONTRIBUTION:

AM treated the patients and was involved in drafting the manuscript. ND was involved in drafting and editing the manuscript. All authors read and approved the manuscript. JFT, KK and EV were involved in the surgical amangement of the patients.

ACKNOWLEDGEMENTS: None
